# Clinical efficacy and safety of spironolactone in patients with resistant hypertension: A systematic review and meta-analysis

**DOI:** 10.1097/MD.0000000000021694

**Published:** 2020-08-21

**Authors:** Cong Chen, Xue-Ying Zhu, Dong Li, Qian Lin, Kun Zhou

**Affiliations:** a Dongzhimen Hospital Beijing University of Chinese Medicine, Beijing; b Shandong University of Traditional Chinese Medicine; c Dongfang Hospital, Beijing University of Chinese Medicine, Beijing, China.

**Keywords:** meta-analysis, resistant hypertension, spironolactone

## Abstract

**Background:**

We conducted a meta-analysis to summarize all available evidence from randomized controlled trial studies regarding the clinical efficacy and safety of spironolactone in patients with resistant hypertension (RH) and provided a quantitative assessment.

**Methods:**

A systematic search of PubMed, Web of Science, Cochrane Library, Embase, and China National Knowledge Infrastructure (CNKI) databases through December 8, 2019, was performed. Randomized controlled trials randomized controlled trials meeting inclusion criteria were included to assess the effect of the addition of spironolactone on office blood pressure (BP), 24-hour ambulatory BP or adverse events in RH patients.

**Results:**

Twelve trials, which enrolled a total of 1655 patients, were included in this meta-analysis. In comparison with placebo, spironolactone significantly reduced office BP (office SBP, weighted mean difference [WMD] = −20.14, 95% CI = −31.17 to −9.12, *P* < .001; office DBP WMD = −5.73, 95% CI = −8.13 to −3.33, *P* < .001) and 24-hour ambulatory BP (ASBP, WMD = −10.31, 95% CI = −12.86 to −7.76, *P* < .001; ADBP, WMD = −3.94, 95% CI = −5.50 to −2.37, *P* < .001). Compared with alternative drugs, spironolactone treatment in RH patients significantly decreased 24-hour ambulatory BP (ASBP, WMD = −6.98, 95% CI = −12.66 to −1.30, *P* < .05; ADBP, WMD = −3.03, 95% CI = −5.21 to −0.85, *P* < .001).

**Conclusion:**

This meta-analysis fully evaluated the antihypertensive effect of spironolactone compared with placebo, alternative drugs, renal nerve denervation and no treatment. Spironolactone can result in a substantial BP reduction in patients with RH at 3 months.

## 1 Introduction

Patients with hypertension are at higher the risk of cardiovascular disease and stroke.^[1]^ Although many antihypertensive drug options are available, the blood pressure (BP) targets for individuals with suboptimal BP are hard to achieve. The inability to reach standard BP levels (below 140/90 mm Hg) despite the concurrent use of 3 or more antihypertensive agents of different classes that include at least one kind of diuretic is defined as resistant hypertension (RH). The etiology of RH is poorly understood, but it is associated with diabetes, chronic kidney disease (CKD), and obesity.^[2]^ To improve BP control in patients with RH, lifestyle modification should be recommended. Despite the ineffectiveness of the first 3 recommended drugs, including calcium channel blockers (CCBs), angiotensin-converting enzyme inhibitors/angiotensin receptor blockers (ACEIs/ARBs), and thiazide diuretics,^[3]^ adherence should be carefully assessed to exclude pseudo resistance. New treatment strategies must be developed to improve BP control.

Currently, the most suitable fourth drug to add to the 3 commonly prescribed antihypertension drugs is not well established.^[4,5]^ However, some recent observational studies have demonstrated that the addition of spironolactone to triple-drug therapy was the most effective add-on drug for patients with RH.^[6]^ As a mineralocorticoid receptor antagonist, spironolactone has a promising effect on lowering BP in hypertensive patients.^[7]^ The adverse effects of spironolactone, including gynecomastia, breast discomfort, and biochemical abnormalities, are relatively.^[6]^ Although some randomized controlled trials (RCTs) have demonstrated the efficacy of spironolactone as a fourth-line therapy for patients with RH, there are no available high-quality, large-scale clinical trials to evaluate the efficacy and safety of spironolactone for RH.^[8]^

We conducted a meta-analysis to summarize all available evidence from RCT studies regarding the clinical efficacy and safety of spironolactone in patients with RH, and we conducted a quantitative assessment. The results of this study were merged with updated available evidence to fully assess the influence of spironolactone on BP compared with placebo and other active interventions. To our knowledge, this was the first study that included data describing BP changes over time.

## 2 Methods

This meta-analysis was performed, according to the Preferred Reporting Items for Systematic Reviews and Meta-Analyses (PRISMA)^[9]^ guidelines, to elaborate the effect of spironolactone on RH.

### 2.1 Data source and search strategy

Two reviewers systematically searched 5 electronic databases, including PubMed, the Web of Science, the Cochrane Library, Embase, and China National Knowledge Infrastructure (CNKI), until December 8, 2019. The search terms were “spironolactone” and “resistant hypertension” matched with “Randomized Controlled Trial” or “Clinical Trial” OR “Controlled Clinical Trial.” The language was not limited. Our meta-analysis selected only the most recent publication when the same study had been published in different journals or in different years. If different studies were performed and published by the same researchers, all studies were selected for the meta-analysis.

### 2.2 Selection criteria

Two reviewers independently examined the titles and abstracts of all obtained articles. Eligible studies met the following inclusion criteria:

(1) RCT design;
(2) patients with RH (the use of at least 3 antihypertensive drugs from 3 different classes did not achieve a standard BP goal);
(3) office, home or ambulatory BP monitoring;
(4) a follow-up of at least 4 weeks; and
(5) a clear description of relevant outcomes.

Articles that were not RCTs, including letters, practice guidelines, conference theses, commentaries, and editorials, were excluded, but relevant articles from the reference lists were retrieved to identify potential studies.

### 2.3 Data collection and quality assessment

Relevant data were collected independently by 2 reviewers, and the third reviewer was responsible for resolving discrepancies through discussion. The standardized predefined form used included 4 parts:

(1) basic information: title, first author, the year of publication, and corresponding address;
(2) study characteristics: the type of intervention and control, daily dosage of intervention, study design, inclusion and exclusion criteria, and RH definition;
(3) the characteristics of participants: sample size, sex distribution, mean age and race; and
(4) results of the study: outcome measures of BP and heart rate. When there were missing data, errors or ambiguous information in the included studies, we contacted the original authors of the study. If the author failed to answer the questions, insufficient information was neglected, or the study was excluded. Different opinions between reviewers were resolved by communication with other reviewers.

We assessed the quality of the involved RCTs by using the Cochrane Collaboration tool. Two reviewers who performed data extraction separately evaluated the risk of bias in the involved studies. Disagreements in scoring were resolved by discussion among the reviewers. Several sources of bias are evaluated in the Cochrane Collaboration tool, including random sequence generation; allocation concealment; the blinding of participants, personnel, and outcome assessment; incomplete outcome data; selective reporting; and anything else, ideally prespecified, and others.

### 2.4 Data synthesis and statistical analysis

All statistical analyses were performed using Cochrane Program Review Manager Version 5.3 (Cochrane Collaboration, Oxford, UK). We used the I2 test and chi-squared test to evaluate the heterogeneity of the included RCTs to estimate the discrepancy across studies (when I2 values <50% and *P* > .10, there was no obvious heterogeneity). A random-effects model was utilized when heterogeneity was obvious; otherwise, a fixed-effects model was used. The risk ratio and weighted mean difference (WMD) were used to represent binary variables and continuous variables, respectively, with a 95% confidence interval (CI). A funnel plot was used to detect publication bias and meta-regression analysis was used to evaluate the factor of effect on heterogeneity.

### 2.5 Grading the quality of evidence

The quality of evidence for outcomes will be evaluated using the Grading of Recommendations Assessment, Development, and Evaluation (GRADE). In the GRADE system, the quality of evidence will be classified into very low, low, moderate, or high judgment.

### 2.6 Ethics

Ethical approval was not required.

## 3 Result

### 3.1 Literature selection and study characteristics

**Figure 1:**
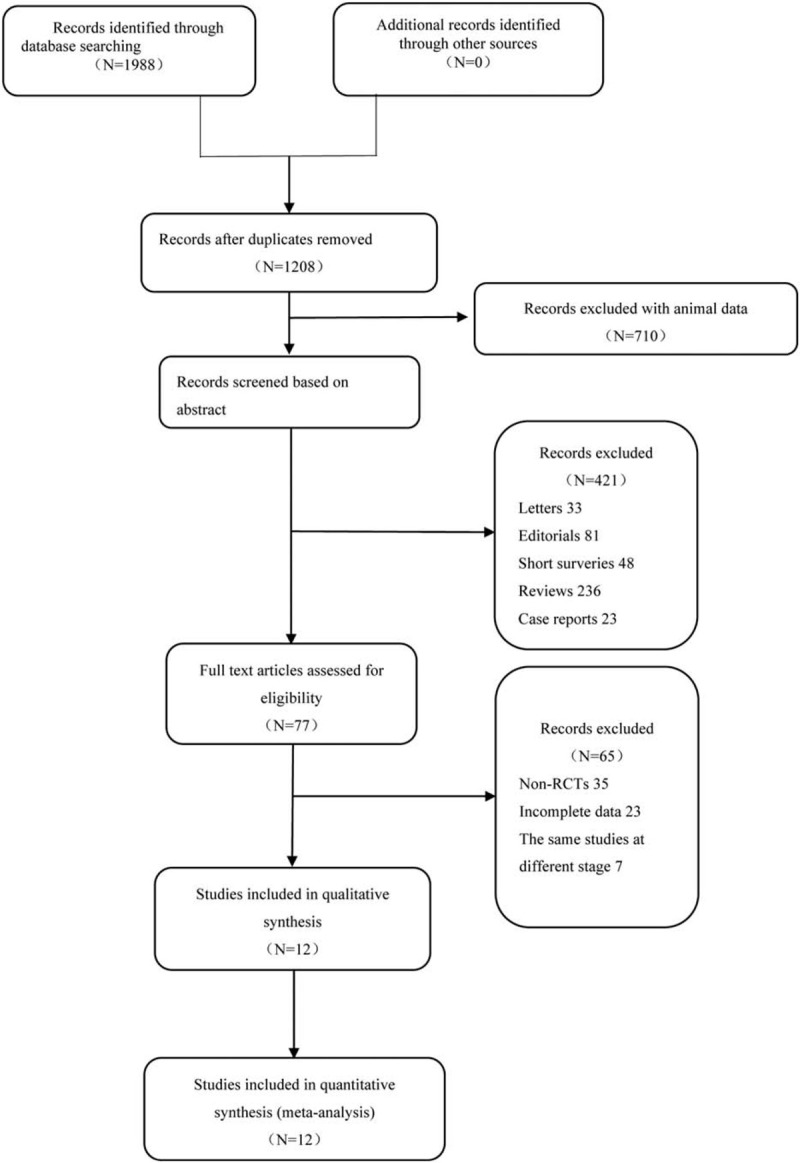
Flowchart demonstrating the study selection process for the meta-analysis.

Overall, 1988 studies were identified by searching databases (296 from PubMed, 1058 from Embase, 474 from the Web of Science, 158 from the Cochrane Library, and 2 from CNKI). After removing duplicates, 1208 remained and were evaluated. By assessing abstracts and full texts, 77 studies were eligible and then further examined thoroughly. Finally, 12 studies met our inclusion criteria, and 65 were excluded for the following reasons: 35 were not RCTs, 23 had incomplete data, and 7 were the same studies at different stages. The literature search processing and reasons for exclusion are listed in Figure 1.

The main characteristics of the included RCTs are listed in Table 1. The major baseline characteristics of each study's spironolactone group and placebo or active control group were similar. We systematically analyzed 12 RCTs, encompassing 1655 patients. There were four RCTs^[10–13]^ that selected patients with CKD, heart failure and obstructive sleep apnea and those on dialysis. Two RCTs^[14,15]^ enrolled participants with type 2 diabetes mellitus and 2 RCTs^[16,17]^ were conducted in patients with true RH.

**Table 1:**
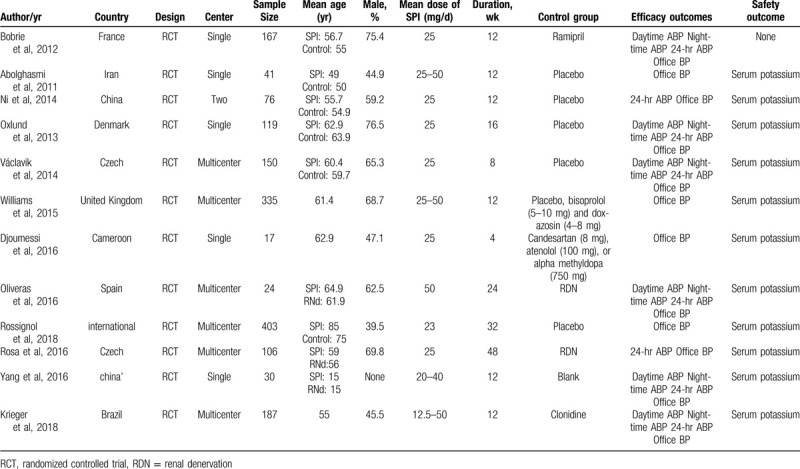
Characteristics of included studies.

### 3.2 Quality assessment

**Figure 2:**
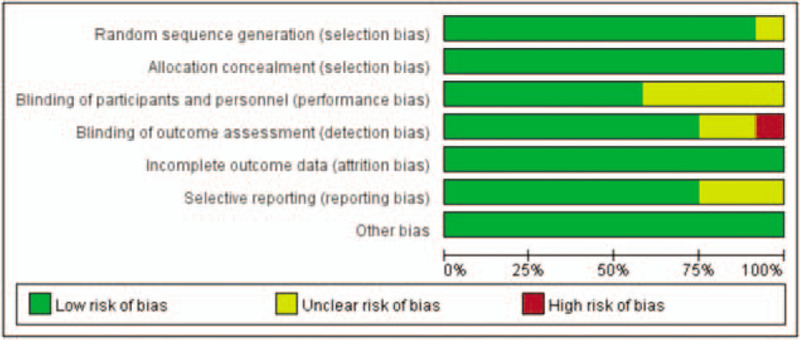
Risk of bias graph.

**Figure 3:**
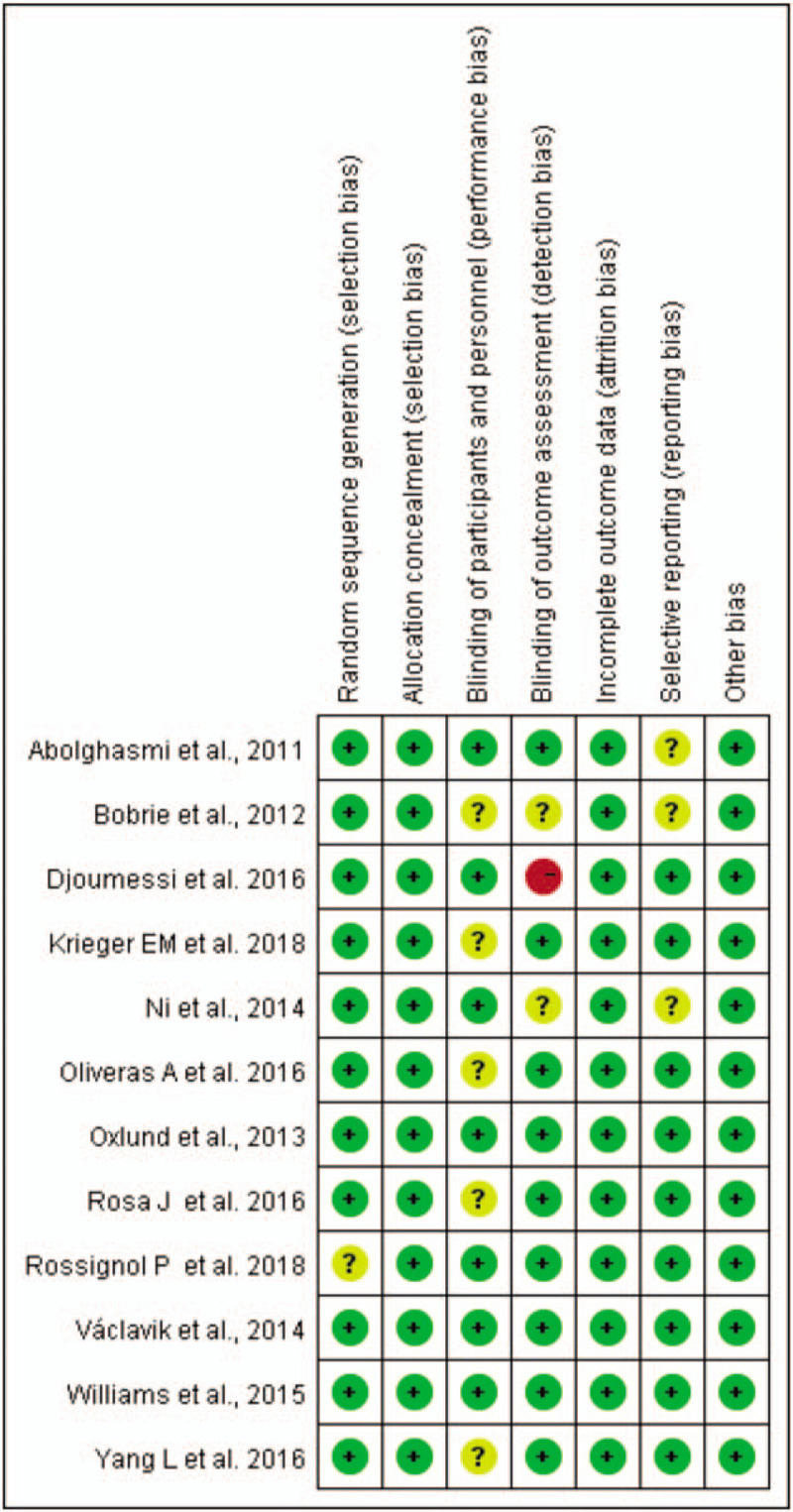
Risk of bias summary.

According to the Cochrane risk of bias tool, most items of the included studies are given in Figures 2 and 3. In these included RCTs, 5 studies^[12,16–19]^ were open label and did not provide clear information on the accurate assessment of the risk of bias; therefore, they were evaluated as having an unclear risk of bias. The details for the blinding of outcome assessment in 1 study were inaccessible; therefore, the study was evaluated as having a high risk of bias.^[14]^ The results of the quality evaluation of the eligible RCTs are shown in Figures 2 and 3.

### 3.3 SBP

**Figure 4:**
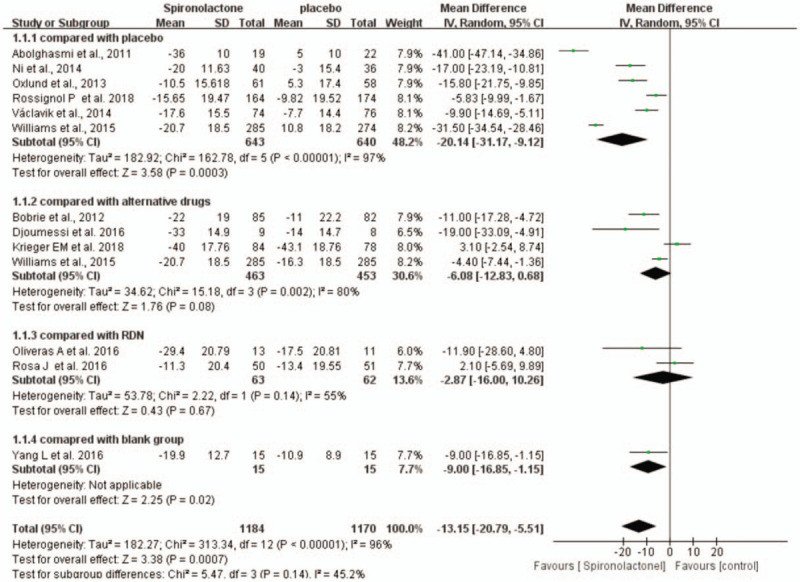
Forest plots comparing the office SBP between the spironolactone group and other groups.

In this meta-analysis, we used a random-effects model to analyze the outcomes. In comparison with placebo, spironolactone significantly reduced office SBP in patients with RH (WMD = −20.14, 95% CI = −31.17 to −9.12, *P* < .001). Compared with no treatment, spironolactone also significantly reduced SBP (WMD = −9, 95% CI = −16.85 to −1.15). However, there was no significant difference between spironolactone and alternative drugs or renal nerve denervation (RND) in the effect on office SBP. Relatively high heterogeneity was shown in comparison with the placebo group, which might be related to the background therapy, the dose of spironolactone, the duration of the intervention and demographic characteristics in each study. (Fig. 4)

**Figure 5:**
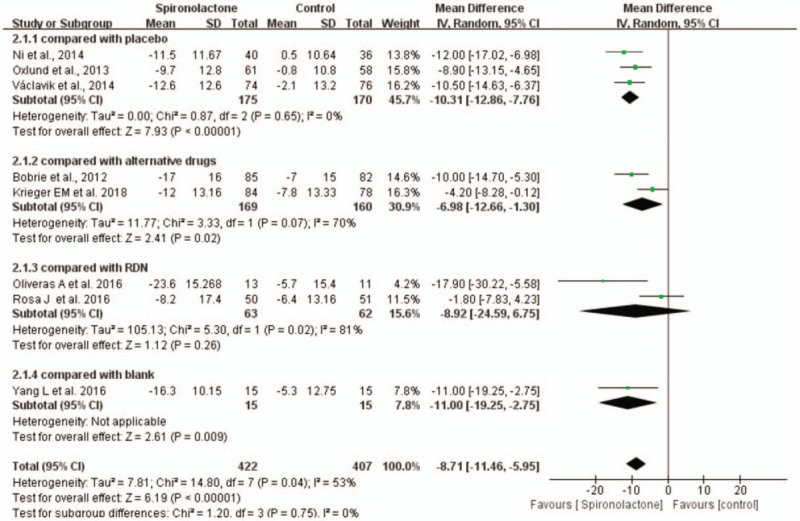
Forest plots comparing the 24-hour ambulatory SBP between the spironolactone group and other groups.

Compared with placebo and no treatment, spironolactone significantly reduced the 24-hour ambulatory SBP (WMD = −10.31, 95% CI = −12.86 to −7.76, *P* < .001) (WMD = −11.00, 95% CI = −19.25 to −2.75). There was no significant difference between spironolactone and RND in the reduction in 24-hour ambulatory SBP. However, in comparison with alternative drugs, spironolactone showed a significant difference in the reduction in 24-hour ambulatory SBP (WMD = −6.98, 95% CI = −12.66 to −1.30, *P* < .05), with relatively obvious heterogeneity (Fig. 5).

### 3.4 DBP

**Figure 6:**
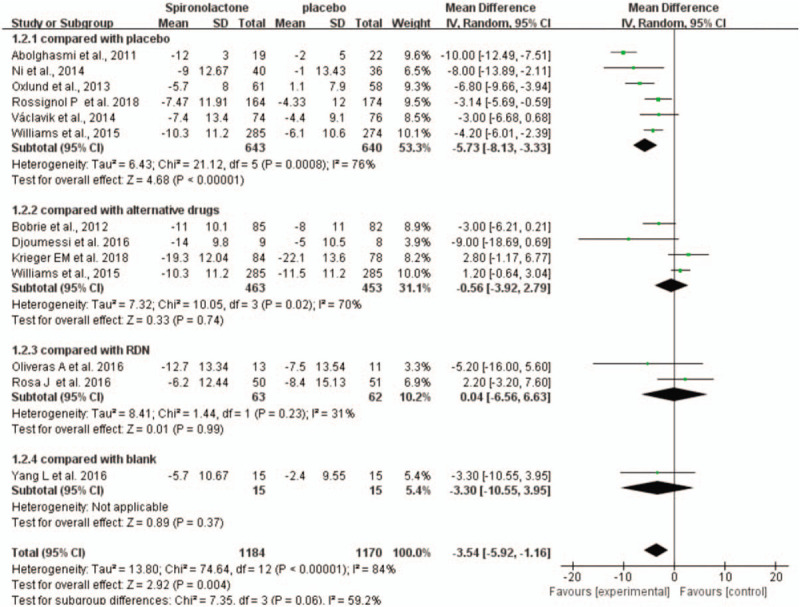
Forest plots comparing the office DBP between the spironolactone group and other groups.

For the changes in DBP from baseline to study endpoint, we performed a random-effects model to analyze the outcomes. Compared with the placebo group, spironolactone significantly decreased office DBP (WMD = −5.73, 95% CI = −8.13 to −3.33, *P* < .001). Apart from comparison with the placebo group, the data analysis did not show any significant difference between spironolactone and the other control groups. Significant heterogeneity was present between spironolactone and placebo because of the dose of spironolactone, the duration of intervention and demographic characteristics (Fig. 6).

**Figure 7:**
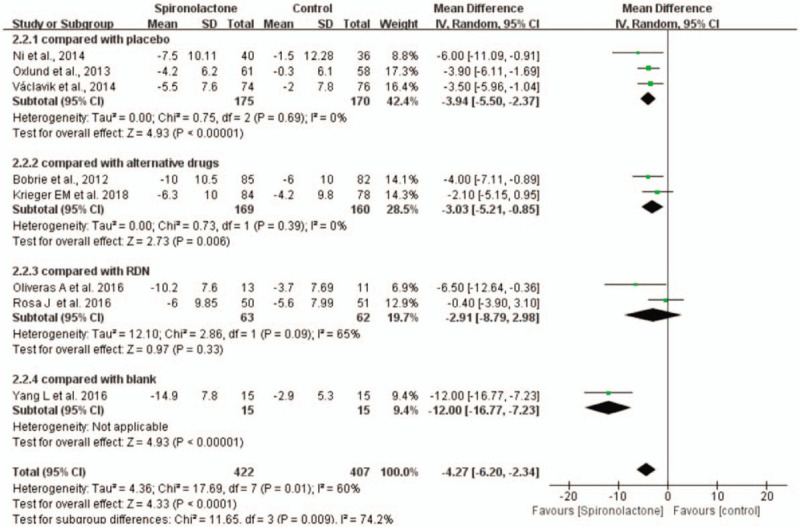
Forest plots comparing the 24-hour ambulatory DBP between the spironolactone group and other groups.

Spironolactone still significantly reduced 24-hour ambulatory DBP compared with placebo (WMD = −3.94, 95% CI = −5.50 to −2.37, *P* < .001) and blank groups (WMD = −12, 95% CI = −16.77 to −7.23). There was no significant difference between spironolactone and RND in the reduction in 24-hour ambulatory DBP. However, in comparison with alternative drugs, spironolactone showed a significant difference in the reduction in 24-hour ambulatory DBP (WMD = −3.03, 95% CI = −5.21 to −0.85, *P* < .001) without heterogeneity (Fig. 7).

### 3.5 The duration of intervention

**Figure 8:**
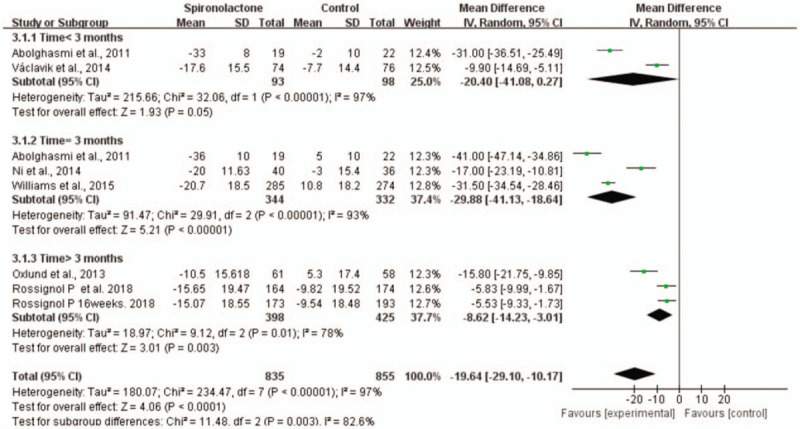
Forest plot of SBP in subgroup analysis defined by the duration of intervention.

**Figure 9:**
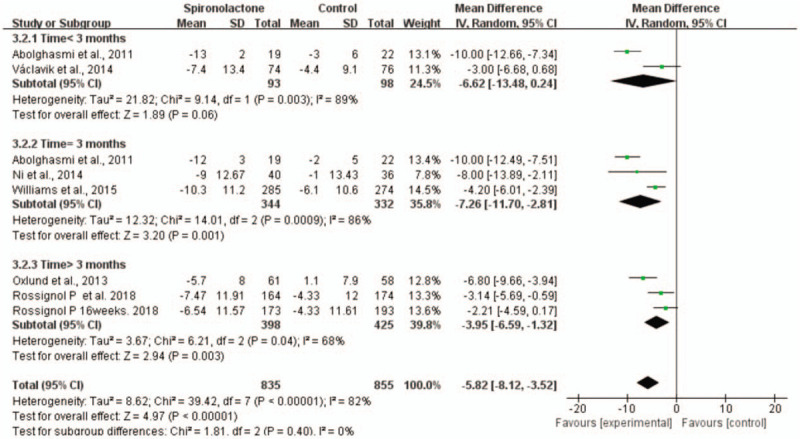
Forest plot of DBP in subgroup analysis defined by the duration of intervention.

In addition, we performed subgroup analysis according to the duration of intervention. In comparison with placebo, the effect of 3 months of spironolactone (WMD = −29.88, 95% CI = −41.13 to −18.64, *P* < .001) was better than that of less than 3 months (WMD = −20.4, 95% CI = −41.08 to −0.27, *P* = .05) or longer than 3 months on SBP (WMD = −8.62, 95% CI = −14.23 to −3.01, *P* < .05). (Fig. 8) Subgroup analysis showed that the effect of 3 months of spironolactone (WMD = −7.26, 95% CI = −11.7 to −2.81, *P* < .001) was better than that of less than 3 months (WMD = −6.62, 95% CI = −13.48 to 0.24, *P* = .06) or longer than 3 months on DBP (WMD = −3.95, 95% CI = −6.59 to −1.32, *P* < .05) (Fig. 9).

### 3.6 Safety outcomes

**Figure 10:**
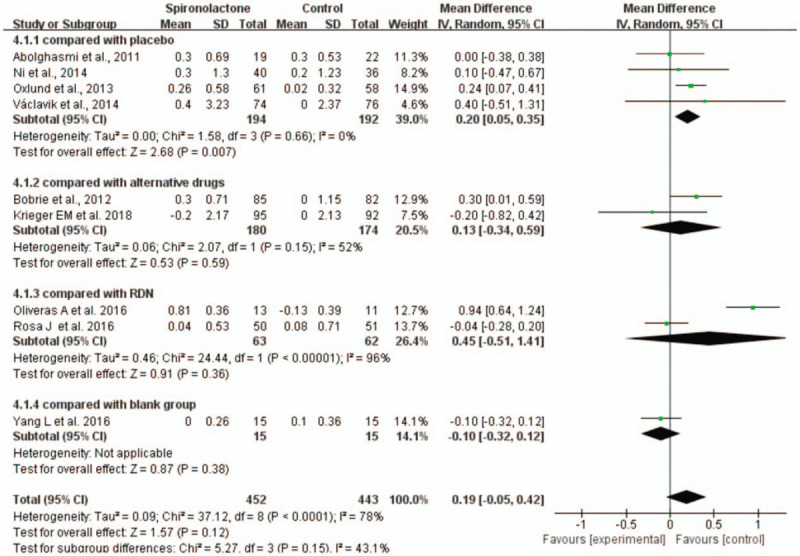
Forest plots comparing the serum potassium levels between the spironolactone group and other groups.

The mean change in serum potassium was defined as the safety outcome, and data were collected from baseline to study endpoint to evaluate whether spironolactone increased the risk of hyperkalemia in patients with RH. Compared to the placebo, spironolactone indeed elevated serum potassium levels (WMD = 0.2, 95% CI = 0.05 to 0.35, *P* < .01) without heterogeneity. However, the spironolactone group did not show any increased risk of elevated serum potassium levels compared with the other-treatment control groups, such as those who received alternative drugs, no treatment, or RND (Fig. 10).

### 3.7 Publication bias

**Figure 11:**
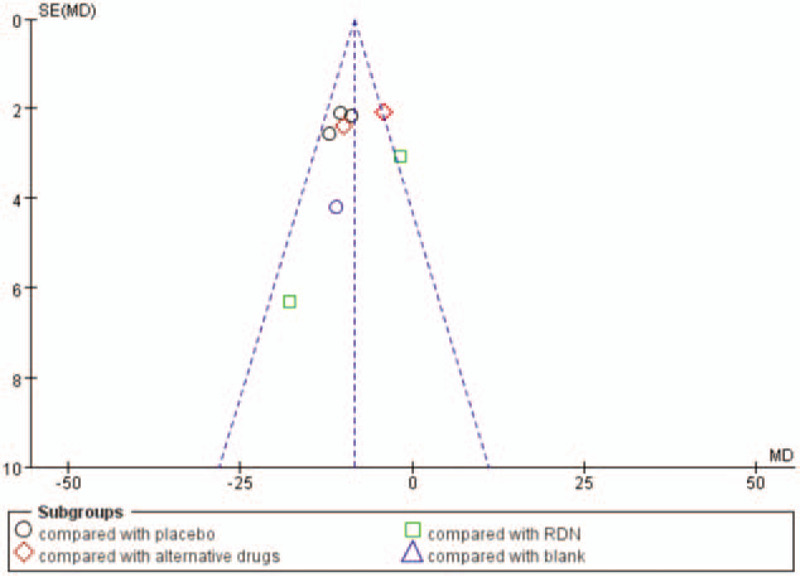
Funnel plot of comparison: spironolactone vs placebo, alternative drugs, RND and no treatment for the outcome 24-hour ambulatory SBP.

A forest plot of the comparison of spironolactone vs placebo, alternative drugs, RND and no treatment for the outcome of 24-hour ambulatory SBP is shown in Figure 11.

### 3.8 Meta-regression analysis for heterogeneity

The following specific variables were separately evaluated for their effects on heterogeneity: country, year, sample size. Meta-regression showed that these variables did not explain heterogeneity observed for specificity (Table 2).

**Table 2:**
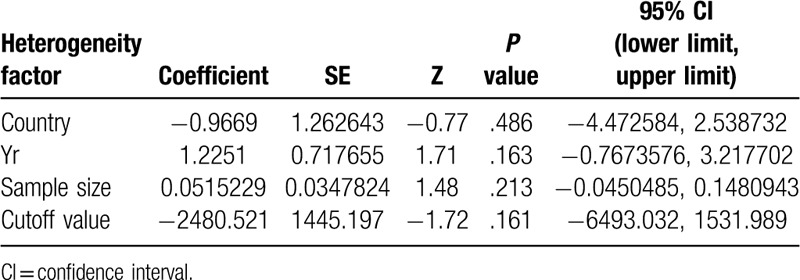
Meta-regression analysis of potential sources of heterogeneity.

### 3.9 The quality of the evidence

The quality assessment of evidence for office BP and ambulatory BP is presented in Tables 3 and 4 using GRADE system.

**Table 3:**
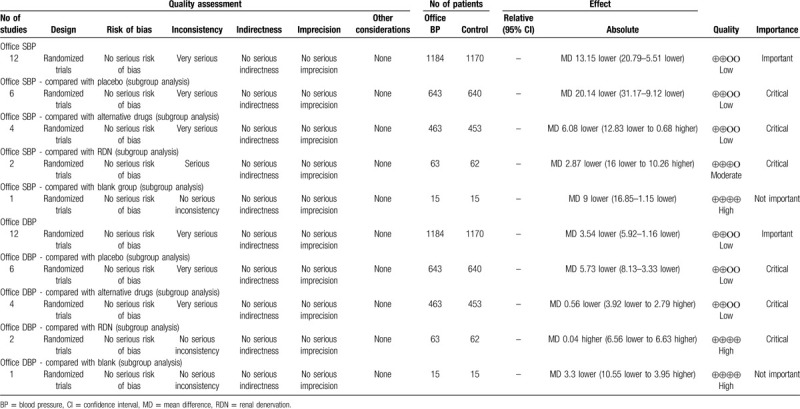
The quality of the evidence: office BP between the spironolactone group and other groups.

**Table 4:**
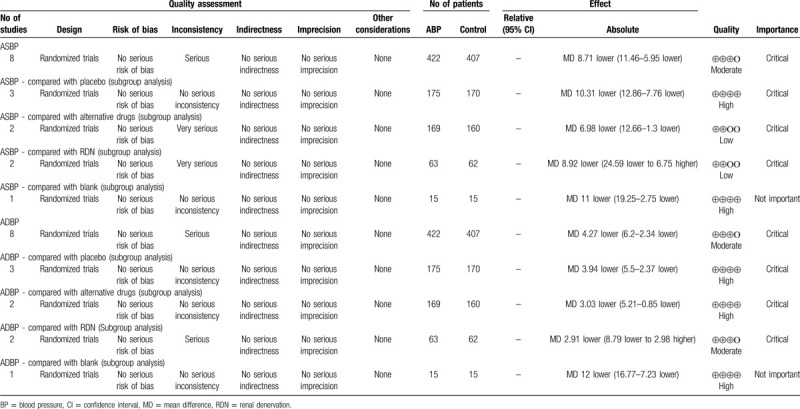
The quality of the evidence: ambulatory BP between the spironolactone group and other group.

## 4 Discussion

The objective of this meta-analysis was to evaluate and quantify the effect of spironolactone on BP in patients with RH. Although the underlying mechanism of RH is unclear, there is numerous evidence that resistant HTN is generally volume-dependent, attributable to differing levels of aldosterone excess with its attendant renal effects on sodium and fluid retention. Such aldosteronism most commonly exists separately from and independent of angiotensin II.^[20]^ The aldosterone-induced volume excess is placed at the root of the development of RH in a large number of patients, with primary aldosteronism being present in approximately 20% of patients.^[21]^ Some studies reported that aldosterone could predict new hypertension, type 2 diabetes mellitus, central obesity, and the use of lipid-lowering drugs in the general community and remained associated with hypertension, obesity, and CKD over 4 years.^[22]^ The administration of spironolactone, a mineralocorticoid receptor antagonist, as a fourth-line agent significantly decreased office BP and 24-hour ambulatory BP compared with placebo based on the results of our meta-analysis. A previous study showed that the add-on use of spironolactone in patients with RH compared with placebo was effective in lowering SBP and DBP.^[23]^ Our subgroup meta-analysis further showed that 3 months of intervention with spironolactone could reduce SBP significantly compared with placebo but that the degrees of reduction would decrease when the intervention was less than 3 months or more than 3 months. This finding that a 3-month duration of intervention with spironolactone had the best effect was newly discovered through our meta-analysis, but the specific mechanism is not clear. Our later research will further explore the underlying mechanism.

This meta-analysis included 4 RCTs (with 916 patients) that evaluated the effectiveness of spironolactone (12.5–50 mg/d) on RH with a control treatment—ramipril in 1 trial, bisoprolol in 1 trial, clonidine in 1 trial and an alternative treatment (candesartan, atenolol, or alpha methyldopa) in 1 trial. The follow-up periods ranged from 8 to 12 weeks. Although there were no significant differences in the effects of spironolactone vs alternative drugs on office SBP and DBP, the results of 24-hour ambulatory BP still demonstrated that spironolactone had a better antihypertensive effect. ABPM has the advantage of less bias than office BP and provides a complete picture of BP values throughout the day. Some studies have supported the concept that ABPM is less vulnerable to other factors effects since it is assessed in a patient's daily life.^[24]^ The area under the curve of the ABPM is more accurate than that of office BP taken at fixed times. When office BP readings fail to demonstrate differences, differences in BP may be detected by ABPM.^[25]^ In the current study, no such differences were noted by office BP, although there was a better antihypertensive effect for ABPM reduction in the spironolactone group compared with the alternative drug group.

This meta-analysis also included 2 trials (with 165 patients) that evaluated the effectiveness of spironolactone and RND. Subgroup meta-analysis showed that spironolactone had the same antihypertensive effect as RND. In renal RND, patients undergo splanchnic sympathectomy for the treatment of severe hypertension. The excess risk associated with RH is importantly linked to the development of CKD, which is known to be a major cardiovascular risk factor. Several studies initially reported that this intervention substantially reduced the elevated BP of RH patients, but this conclusion has not been supported by the results of this meta-analysis. The antihypertensive efficacy and overall validity of RND did not show a difference compared with spironolactone. In addition, many Chinese people tend to reject invasive treatment strategies.^[26]^ Based on our results, spironolactone had a better antihypertensive effect than other drugs, and its effects were not worse than those of RND, making it more suitable for many Chinese individuals.

Our study showed a significant benefit of spironolactone use in lowering BP in patients with RH, and the most frequent adverse events, such as hyperkalemia and gynecomastia/mastodynia, should be considered.^[27]^ Our subgroup meta-analysis showed that spironolactone significantly increased the concentration of serum potassium compared with placebo. When compared with the other interventions, there was no significant difference in the serum potassium concentration with the use of spironolactone. The mild degree of the elevation of serum potassium with the administration of spironolactone may indicate the safety of the intervention. Although we did not have direct data to assess the incidence of adverse events, hyperkalemia still occurred in ∼3% of patients receiving spironolactone in another study.^[28]^ Therefore, it is necessary to pay attention to serious adverse events caused by spironolactone.

### 4.1 Limitations

Several limitations of this study should be mentioned. First, unexplained high heterogeneity might affect the results of the subgroup meta-analysis, although we used strict inclusion criteria to ensure the inclusion of high-quality RCTs. Second, we included several trials with small sample sizes, which may have resulted in a risk of bias in this meta-analysis. Third, original data could not be accessed for every study, which might influence the results, although this is a limitation inherent to all study-level meta-analysis. Fourth, we did not evaluate the incidence of hyperkalemia due to a lack of data in multiple studies. In the future, the occurrence of other long-term complications in patients who use spironolactone should be analyzed. Fifth, the prevalence of primary aldosteronism (PA) is very high in RH patients. However, several studies did not evaluate the influence of spironolactone on PA. Therefore, we did not fully evaluate the safety of using spironolactone.

### 4.2 Future directions

The finding that a 3-month duration of spironolactone intervention had the best effect was newly discovered through our meta-analysis, but the specific mechanism is not clear. Our later research will further explore the specific mechanism of the drug to provide theoretical support for the clinical application of spironolactone.

## 5 Conclusion

This meta-analysis fully evaluated the antihypertensive effect of spironolactone compared with placebo, alternative drugs, RND and no treatment. Spironolactone can result in a substantial BP reduction in patients with RH at 3 months. Additionally, more large and long-term randomized controlled trials, including data that evaluate the incidence of hyperkalemia and long-term adverse events, are needed.

## Author contributions

XXXX.
